# The alliance-outcome association in borderline and obsessive-compulsive personality disorder

**DOI:** 10.3389/fpsyt.2023.1094936

**Published:** 2023-03-08

**Authors:** Peter Beiling, Susan Schurig, Andrea Keller, Kerstin Weidner, René Noack

**Affiliations:** TU Dresden, Carl Gustav Carus Faculty of Medicine, Department of Psychosomatic Medicine and Psychotherapy, Dresden, Germany

**Keywords:** borderline personality disorder, obsessive-compulsive personality disorder, therapeutic alliance, psychotherapy outcome, prediction

## Abstract

Personality disorders are considered a possible factor affecting the relationship between therapeutic alliance and therapy outcome. The present study investigated the alliance-outcome effect in patient groups with borderline personality disorder (BPD) and obsessive-compulsive personality disorder (OCPD). Data derived from a sample of *n* = 66 patients, treated in a day care hospital setting with a dialectical-behavioral and schema therapeutic treatment concept. Patients rated their symptom severity at admission, early alliance after 4–6 therapy sessions and symptom severity as well as alliance at discharge. Results showed no significant differences between BPD and OCPD patients regarding symptom severity and alliance. Multiple regression analyses indicated that the alliance was a significant predictor of symptom reduction, however only in the OCPD group. Our results showed an exceptionally strong alliance-outcome relationship in OCPD patients, suggesting that focusing on building a strong alliance and measuring it early in therapy may be especially beneficial for this patient group. For patients with BPD, a more regular screening of the therapeutic alliance might be helpful.

## 1. Introduction

The therapeutic alliance has been intensively researched for decades with regard to its determinants and its influence on therapeutic success. Bordin's pantheoretic conceptualization (1) defines the therapeutic alliance as an agreement between therapist and patient regarding goals, the tasks necessary to achieve them as well as an overall positive affective relationship toward one another.

Meta-analyses have documented a small to medium, yet robust, positive correlation between the therapeutic alliance and treatment outcome, which is evident across different types of psychotherapy, therapy settings, and assessment instruments (2–4). Previously, however, the causal direction of the correlation has been repeatedly questioned (5–8). In recent years, studies with settings that allow more precise statements about the causal direction (e.g., through session-to-session measurements of alliance and outcome) have strengthened the assumption of a causal influence of therapeutic alliance on treatment outcomes (9, 10). However, a complex, reciprocal process can be assumed whereby the therapeutic alliance promotes therapeutic progress while therapeutic progress simultaneously positively influences he therapeutic alliance (11–14).

Little is known about possible moderators of the alliance-outcome relationship and the exact mechanisms by which the therapeutic alliance influences therapy outcome. It can be assumed that several factors pertaining to the patient, the therapist, as well as factors relating to the setting are involved in this process. Previous studies have identified several patient-related moderators of the alliance-outcome relationship, amongst others severity of symptoms at baseline (14), number of previous depressive episodes (15, 16), gender (17), ethnicity (18), attachment-associated variables (19, 20), diagnosis (2), and life satisfaction as a within client effect (21). Research has also supported the importance of therapist contributions for improved patient outcomes (22). Finally, aspects of the therapy setting that have been found to have an effect are duration of therapy (14), time of assessment, and outcome measure (3). Taking this complexity of factors into account could explain the great variance found in the size of the alliance-outcome relationship across studies (2–4).

Whether a patient has been diagnosed with a personality disorder represents another patient-related factor that may impact the alliance-outcome relationship. Only few studies have examined the role personality disorders play in this regard and these investigations have yielded inconsistent findings to date. While some studies found the alliance-outcome effect to be stronger in patients with personality problems as compared to patients without personality problems (9, 23), a meta-analysis by Del Re et al. (22) showed no correlation between the extent of the alliance-outcome effect and the proportion of patients with personality disorders in the studies included. A possible explanation for these inconsistencies is that the effect of the therapeutic alliance on treatment outcome could vary depending on the type of personality disorder, i.e., between patient groups with different personality disorder diagnoses.

When comparing the different types of personality disorders, differences as well as similarities become apparent. A basic commonality of all personality disorders is their definition in the Diagnostic and Statistical Manual of Mental Disorders (DSM-5) (24) as an inflexible pattern of inner experience and behavior that deviates significantly from the expectations of the socio-cultural environment, is manifested during adolescence and is relatively stable over time. Interpersonal dysfunctionality in particular has been characterized as a central aspect of personality disorders (25), thereby highlighting the importance of investigating how personality disorders influence the effect the therapeutic alliance has on therapy outcome. It is assumed that interpersonal dysfunctionality impedes the formation of a helpful and stable therapeutic relationship, at least in case of some personality disorders (26). Nevertheless, a recent meta-analysis by Flückiger et al. (2) showed no overall difference in the quality of the therapeutic alliance between patients with and patients without the diagnosis of a personality disorder. However, the study did show that the alliance-outcome effect varied greatly in samples of patients with borderline personality disorder, thereby suggesting that the dynamics of the alliance-outcome relationship may vary between personality disorder types after all.

In the present study, patient groups with borderline personality disorder (BPD) and obsessive-compulsive personality disorder (OCPD) will be examined for possible differences in their therapeutic alliance and alliance-outcome relationship. BPD and OCPD appear particularly suitable for this differential analysis of personality disorders as they belong to different DSM-5 clusters of personality disorders and are among the most common personality disorders in outpatient groups (27). Moreover, BPD and OCPD also represent the most frequently occurring personality disorders in our clinical sample, which highlights their high relevance in everyday clinical practice.

Borderline personality disorder is characterized by a pattern of instability in self-image, interpersonal relationships, and affects (DSM-5) (24). Common symptoms are increased impulsiveness, manipulativeness, self-harm, difficulties controlling anger, and chronic feelings of emptiness. A rapid change between fear of abandonment and thus strong dependency in relationships as well as intense rejection and devaluation are typical amongst BPD patients. Compared to nonclinical individuals BPD patients tend to show greater intraindividual variability as well as more extremes in their mood and interpersonal behavior (28). Following the DSM-5 (24) classification of personality disorders into clusters, BPD forms part of cluster B, which histrionic, antisocial, and narcissistic personality disorder are also part of.

In contrast, a pattern of preoccupation with orderliness and details, perfectionism, self-control, interpersonal control, and a lack of flexibility is characteristic of OCPD. Typical symptoms include excessive devotion to work, over-conscientiousness, in ability to discard worthless objects, difficulties delegating tasks, miserliness, and rigidity. OCPD is assigned to cluster C of the DSM-5, along with avoidant personality disorder and dependent personality disorder (24).

Only few studies empirically comparing BPD and OCPD exist. Patients with BPD have been found to differ from patients with OCPD regarding several aspects of attachment style, such as being more likely to show angry withdrawal and compulsive careseeking attachment patterns (29). A study by Skodol et al. (30) showed that patients with BPD tended to have greater impairment at work, in social relationships, and at recreational activities than patients with OCPD (30). However, Soeteman et al. (31) found no differences in the quality of life between patients with BPD and OCPD.

Considering the core characteristics of BPD and OCPD described above, it seems plausible to assume that different challenges with regard to the therapeutic alliance may exist for these patient groups. On the one hand, OCPD patients may show a strong reticence to engage emotionally in the therapeutic alliance and have problems in empathic perspective taking (32). Meanwhile, BPD patients tend to oscillate rapidly between attachment and disengagement. Consequently, their desire for great closeness can easily be interfered with, for instance through feelings of rejection, thereby leading to ruptures in the alliance linked with angry withdrawal behavior, resulting in an overall decreased likelihood of engagement in treatment (29, 33, 34).

The aim of the present study is to obtain a more differentiated understanding of the importance of the therapeutic alliance and its measurement in the successful treatment of patients with different personality disorders.

In a first step, we descriptively examined possible group differences in the quality of the therapeutic relationship and the symptom burden between BPD and OCPD patients. While the situation of research regarding impairment in social relationships and quality of life seems to be inconsistent as described above, we assumed that BPD patients would show higher symptom burden at admission and lower values in therapeutic alliance early in therapy as compared to OCPD (Hypothesis 1). Based on the specific characteristics of BPD and OCPD, their respective challenges with regard to the therapeutic alliance, as well as the rapidly changing nature of the alliance in BPD, we assume that the overall predictive value of the therapeutic alliance, measured once early on in therapy, for treatment outcome will be greater for OCPD patients compared to BPD patients (Hypothesis 2).

## 2. Materials and methods

### 2.1. Participants

Patients were treated in a multimodal, multidisciplinary setting with dialectic behavioral therapy (DBT) and Schema Therapy for an average of 12 weeks. Therapy included individual therapy twice a week (with a total duration of 75 min), interactional group therapy twice a week, as well as Schema Therapy, psychoeducation, mindfulness-based therapy, body psychotherapy, nordic walking and social skills training once a week in a group setting. Patients diagnosed with borderline personality disorder received art therapy once a week while patients diagnosed with obsessive-compulsive personality disorder received music therapy. Schema Therapy was a central element of therapy for both groups. Patients with borderline personality disorder received additional DBT skills group training, if self-harming behavior has occurred repeatedly in the past. Before admission, possible contraindications such as the presence of psychotic disorders or acute suicidal tendencies were examined. In the case of addictive disorders, long-term abstinence was checked in advance of therapy to ensure that only sufficiently abstinent patients began therapy. Data are based on our internal clinical quality management and were gathered using standardized self-rating questionnaires at the time of admission (t_1_), 3 weeks after treatment commencement and approximately after three sessions with the therapist (t_2_), and at discharge (t_3_). Participants provided written informed consent after having been briefed about the scientific use of the data collected. The study was performed according to the Declaration of Helsinki and was approved by the Ethics Committee of the Technische Universität Dresden (EK494122016).

Between 2007 and 2017, a total of *n* = 809 patients with a wide range of different diagnoses were treated in a day-clinical setting. The present study included data from all patients who were diagnosed with borderline personality disorder (BPD) or obsessive-compulsive personality disorder (OCPD) as a primary clinical diagnosis. Out of the *n* = 809 patients, *n* = 102 patients were diagnosed with BPD and *n* = 41 patients were diagnosed with OCPD as the primary clinical diagnosis. In the OCPD sample there was only one case of therapy discontinuation, however this case could still be included in the analysis as all relevant questionnaires were completed. In the BPD sample there were 30 patients who discontinued therapy, 4 of which could be included in the analysis. A total of 26 patients refused to fill out questionnaires at discharge. There was a large drop out of 40 cases in the BPD sample and 11 cases in the OCPD sample due to missing data. The vast majority of this was due to the fact that the questionnaire for the interim survey was temporarily not distributed due to administrative reasons. The final sample consisted of 66 day clinic patients, 36 with BPD and 30 with OCPD as a primary clinical diagnosis. A total of 18 psychotherapists were involved in the treatment of the sample, treating between one and 15 patients.

Table 1 contains comparative data of the subsamples. Statistically significant differences were found in several variables. The BPD subsample was younger (medium effect), more likely to be female (small effect), less likely to be in a stable partnership (small effect), had significantly lower educational attainment (large effect), and shorter treatment duration (medium effect).

**Table 1:** Sociodemographic data and statistical comparison of patients with borderline personality disorder and obsessive-compulsive personality disorder.

**Category**	**BPD**	**OCPD**	**Analyses**
	***n*** **(%)**		
***N***	36	30	
**Age** (in years); mean ± SD range	28.69 ± 6.93 20–48	35.70 ± 10.09 20–48	*U =* 306.5, *Z* = −3.01, *p* = 0.003, *r* = 0.37
**Sex** (female)	33 (91.7)	21 (70.0)	χ ^2^ = 5.16, *p* = 0.02, *Cohen's w* = −0.28
**Partnership** (firm partnership)	19 (52.8)	22 (73.3)	χ ^2^ = 2.94, *p* = 0.09, *Cohen's w* = 0.21
**Education** (at least High School)	3 (8.3)	17 (56.7)	χ^2^ = 18.1, *p < 0.001, Cohen's w* = 0.52
**Existing incapacity for work**	21 (67.7) (*n =* 31)	17 (58.6) (*n =* 29)	χ ^2^ = 0.54, *p* = 0.46
**Duration of incapacity for work** (in weeks); mean ± SD range (n)	14.33 ± 15.00 1–50 (*n =* 15)	26.13 ± 23.94 2–87 (*n =* 16)	*U =* 81, *Z* = −1.54, *p* = 0.13
**Duration of therapy** (in days) mean ± SD range	78.83 ± 14.01 39–102	87.33 ± 5.29 81–95	*U =* 278.5, *Z* = −3.44, *p* = 0.001, *r* = 0.42

Mann–Whitney U-tests and Chi-Square tests were conducted to test for differences between groups regarding the sociodemographic variables.

In the BPD subsample, the most common secondary diagnoses were major depressive disorder, recurrent episode (*n* = 20; 55.6%), social phobia (*n* = 15; 41.7%), anorexia nervosa (*n* = 12; 33.3%), and PTSD (*n* = 9; 25%). In the OCPD subsample, the most common secondary diagnoses were also major depressive disorder, recurrent episode (*n* = 22; 73.3%) and social phobia (*n* = 8; 26.7%) as well as major depressive disorder, single episode (*n* = 6; 20.0%) and obsessive-compulsive disorder (*n* = 5; 16.5%). During therapy, 58.3% of the patients in the BPD sample and 56.7% of the patients in the OCPD sample were under psychopharmacological medication, the vast majority of them received SSRI's, SNRI's or tetracyclic antidepressants.

### 2.2. Material

The diagnosis of BPD or OCPD used for group allocation was a composite clinical judgement based on the clinical impression of the treatment team, the results of the International Personality Disorder Examination (IPDE) (35) and, in cases of uncertainty, an additional SCID II interview (Clinical Interview for DSM–IV Axis II Disorders) (36) conducted by trained interviewers.

The Brief Symptom Inventory-18 (BSI-18) (37) is a widely used self-report questionnaire consisting of 18 items which are rated on a 5-point Likert scale. It assesses psychological distress during the last 7 days with the scales somatization, depression and anxiety. The global severity index can be calculated as a global indicator of psychological stress, thereby permitting comparability of severity of illness across different disorders and settings. The BSI-18 has shown questionable to high internal consistencies (α = 0.63 to α = 0.93), sufficient discriminatory power (*ri* ≥ 0.40), and high convergent validity. In the present study, the BSI-18 was distributed to patients at admission (t_1_) and discharge (t_3_).

The Helping Alliance Questionnaire (HAQ) (38) is a self-report questionnaire assessing the quality of the therapeutic alliance. It consists of 11 items which are rated on a 6-point Likert scale. The HAQ is frequently used and shows adequate construct validity and reliability. In a recent psychometric review by Nübling et al. (39), the reliability and validity analyses showed satisfying to good results. The HAQ was distributed as part of the interim survey 3 weeks after admission (t_2_) and at discharge (t_3_).

### 2.3. Statistical analysis

Statistical analyses were conducted using IBM SPSS Statistics (Version 27). Mean imputation was performed if up to a maximum of 20% of values were missing from a questionnaire. Sensitivity analyses revealed no statistically significant different results when mean imputation was performed compared to when only completely available questionnaire values were included. Hence, mean imputed values were used in subsequent analyses. To test the assumptions of the statistical analyses for the subgroup differences shown in Tables 1, 2, we assessed normal distribution using the Kolmogorov-Smirnov test and Shapiro-Wilk test and homogeneity of variance using Levene's test. Several metric variables (age, duration of therapy, duration of incapacity for work, HAQ at interim survey) were not normally distributed. In these cases, we used the non-parametric Mann–Whitney *U*-test instead of an independent samples *t*-test. To test for group differences regarding frequencies in categorical sociodemographic variables we conducted Pearson's Chi-Squared test. For statistically significant subgroup differences effect size was calculated using r and Cohen's w for the Mann–Whitney U and Chi-Squared test respectively. An effect size of *r* or *w* ≥0.01 is considered a small effect, ≥0.03 is considered a medium effect, ≥0.05 is considered a large effect (40).

**Table 2:** Clinical characteristics of patients with borderline personality disorder and obsessive-compulsive personality disorder.

**Variable**	**BPD (*****N =*** **36)**		**OCPD (*****N =*** **30)**		**Analyses^c^**
	**M** ±**SD**	**Range**	**M** ±**SD**	**Rang** ***e***	
GSI ${\text{t}}_{1}^{\text{a}}$	23.50 ± 12.36	2–62	24.12 ± 12.56	2–51	*t* = −0.202, *p* = 0.84
GSI ${\text{t}}_{3}^{\text{a}}$	16.46 ± 11.37	0–53	15.60 ± 11.45	2–46	*t* = −0.304, *p* = 0.76
GSI difference t_3_ to t_1_	7.04 ± 10.90	−20.00–40.47	8.52 ± 11.33	−20.00 – 30.53	*t* = −0.539, *p* = 0.59
HAQ ${\text{t}}_{2}^{\text{b}}$	51.03 ± 10.44	13–66	54.63 ± 6.20	42–65	*U =* 423, *Z* = −1.51, *p* = 0.13
HAQ ${\text{t}}_{3}^{\text{b}}$	54.83^d^ ± 6.35	43–66	56.49 ± 7.14	42–66	*t* = −0.995, *p* = 0.32

a Global Severity Index (GSI) of the Brief Symptom Inventory (BSI-18) measuring overall psychological symptom stress at admission (t_1_) and discharge (t_3_).

b Helping Alliance Questionnaire (HAQ), self-rating. Quality of therapeutic alliance at interim survey after 3 weeks (t_2_) and discharge (t_3_).

c Independent samples t-tests and Mann-Whitney U-tests were conducted.

d n = 35.

To examine differences in the alliance-outcome relationship, two independent multiple regression analyses were conducted for patients with BPD and patients OCPD respectively. Calculations used a multiple linear regression model and were all adjusted for symptom load (BSI-18) at t_1_, which was included as a control variable in the regression model. Other independent variables included in the regression model as control variables were age, sex, treatment duration, therapy discontinuation, and partnership status. Difference values of the GSI between t_1_ and t_3_ were calculated and used as a measure for symptom reduction. Standardized z-scores were calculated for the GSI at t_1_, the GSI change, and the HAQ values.

## 3. Results

As can been seen in Table 2, there were no statistically significant group differences in GSI and HAQ scores at the different measurement points. Nevertheless, in the BPD group, there was a tendency for the values of the HAQ recorded after 3 weeks of therapy to show a larger standard deviation. The range of HAQ values differed between the BPD and OCPD groups such that lower HAQ values were more present in the BPD group sample than in the OCPD group (see Table 2). We additionally conducted a dependent *t*-test for paired samples which revealed that the GSI score was significantly lower at t_1_ than t_3_ across the entire patient sample (*t* = 5.677, *p* < 0.001).

The results of the two multiple linear regression models presented in Table 3 show that the overall model fit was higher in the OCPD group (*R*^2^ = 0.458) than in the BPD group (R^2^ = 0.125). The therapeutic alliance recorded after 3 weeks emerged as a statistically significant predictor of symptom reduction, however only in the group of OCPD patients (OCPD: β = 0.50; 95% CI [0.20; 0.09]; BPD: β = –0.03; 95% CI [–0.29; 0.25). The control variables (age, sex, treatment duration, therapy discontinuation, and partnership status) had no statistically significant predictive effect on symptom reduction (*p* > 0.05) and were hence not included in the presentation of results in Table 3 for reasons of clarity.

**Table 3:** Results of the multiple linear regression models showing the association between GSI^a^ change and HAQ^b^ score in the borderline and obsessive-compulsive personality disorder subsamples, considering different control variables^c^.

**Group**	**Goodness of fit coefficients**				**Regression coefficients**		**Confidence interval**	
**Variables**	**R** ^2^	**corr. R** ^2^	***F***	***p***	β	***p***	**Lower limit**	**Upper limit**
**BPD**	0.3	0.13	1.71	0.15				
HAQ (t_2_)					−0.03	0.88	−0.29	0.25
GSI (t_1_)					0.56	0.01	0.14	0.96
**OCPD**	0.59	0.46	4.51	0				
HAQ (t_2_)					0.5	0.01	0.2	1.09
GSI (t_1_)					0.65	< 0.001	0.35	0.98

a Global Severity Index of the Brief Symptom Inventory.

b Helping Alliance Questionnaire (HAQ), self-rating. Quality of therapeutic alliance at interim survey after 3 weeks (IS).

c Age, sex, treatment duration, therapy discontinuation and partnership status. None of the control variables had a statistically significant effect and are therefore not presented.

## 4. Discussion

Contrary to our assumption of a higher initial symptom burden in BPD patients, results showed no statistically significant group differences in the GSI values at admission compared to OCPD patients. Similarly, BPD and OCPD patients did not differ significantly in their GSI values at discharge or regarding therapy outcome, which was measured as the change in GSI score from admission to discharge. One possible explanation for the comparably high symptom burden of OCPD patients in our sample could be that the patients in the present sample were preselected to a certain extent on the basis of their symptom severity since intensive day-care therapy would not have been indicated for OCPD patients with only mild symptom burden. Nevertheless, this result points to the importance of not underestimating the extent of impairment in patients with personality disorders commonly considered to be less severe.

Contrary to our expectation, there were no statistically significant differences between the groups regarding the helping alliance scores at the different points of data collection. Results further showed that strongly negatively rated therapeutic alliance only appeared in the BPD group, consequently a slightly higher variance was noticeable amongst BPD patients. However, Levene's test for homogeneity of variance did not reveal any statistically significant group differences, possibly due to limited statistical power. Most importantly, it can be assumed that among patients who dropped out of therapy and therefore could not be included in the analysis, which was more common in the BPD group, poor therapeutic alliance would occur frequently. A meta-analysis by Sharf et al. (41) found a moderately strong relationship (*d* = 0.55) between weak therapeutic alliance and drop-out of psychotherapy. However, with the available data it was not possible to differentiate between patients who discontinued therapy at their own request due to dissatisfaction with the therapeutic alliance and those patients who were discharged by the clinic, for instance due to violations of house rules or severe suicidal symptoms requiring admission to inpatient psychiatric treatment.

The present study showed a statistically significant association between the helping alliance after 3 weeks and therapy outcome, however only in the OCPD group. The association we found in our OCPD group lies significantly above the average reported in recent meta-analyses (2) (*r* = −0.219), (3) (*r* = 0.275), (4) (*r* = 0.22). A possible explanation as to why we found therapeutic alliance to predict treatment outcome only amongst OCPD and not BPD patients could be the different patterns of alliance development characteristic of these personality disorders. Although our data does not allow a deeper analysis of alliance development patterns, it can be assumed that patients in the OCPD group tend to have more linear alliance development patterns while patients in the BPD group may have more rupture resolution patterns as a result of the characteristic interactional problems in BPD (42). Due to the more discontinuous, cyclical nature of rupture resolution patterns, a singular alliance measurement may have lower predictive validity for BPD patients. The interaction styles and difficulties typical of OCPD patients are in comparison steadier in nature, for example rigidity, passive-aggressive styles, and inhibition in emotional expression. Thus, singular measures of alliance could give a more accurate impression of the alliance beyond temporary ruptures in the therapeutic relationship and thus be of higher predictive validity compared to BPD patients. It should also be considered that patients with BPD might benefit more from factors beyond the therapeutic alliance in individual therapy, e.g., psychoeducation on emotion regulation and DBT skills training in group setting.

Our study has several limitations. First and most importantly, the data base we used does not assess therapeutic alliance and symptom burden at sufficient measurement points to allow for a more elaborate session-to-session measurement. Therefore, it was not possible for us to differentiate between within-subject variability and between-subject variability. Hence, we are unable to make a definitive statement about the causal direction of the alliance-outcome effect. Instead, we must base our assumptions and interpretation of findings on the available literature summarized above. Moreover, the investigation of additional sub-components of the alliance-outcome effect could not be disentangled in our study either. Particularly in light of the fact that the alliance-outcome effect is assumed to be composed of a trait-like component, such as patient characteristics, and a state-like component, namely changes in the alliance throughout the therapeutic process, future research should take into account the distinct influences these components have on the alliance-outcome effect (43).

Second, this investigation was limited by its small sample size. As we wanted to analyze the alliance-outcome relationship in patients with specific personality disorders, we were only able to include a very small sub-sample of the complete data set in our analyses. Hence, our findings should be interpreted with caution. Although the results were statistically significant, the small sample size resulted in large confidence intervals which may indicate a rather instable effect with the possibility of a Type II error. Additionally, results may be confounded by the imbalance in drop-out rates, with a higher rate amongst BPD patients than OCPD patients. However, at the cost of a larger sample, we were able to use data from patients with different personality disorder diagnoses to examine the alliance-outcome effect.

Finally, whilst the naturalistic nature of the present study brings the benefit of reflecting everyday clinical practice, the fact that there was no standardized, homogeneous therapeutic process could represent a confounding effect which may have biased our findings. For example, interventions from dialectical behavioral therapy are more frequently used in the treatment of BPD, while schema-therapeutic methods are more commonly indicated in the treatment of patients with OCPD. These differences in therapeutic methods could have influenced patients' experience of the therapeutic alliance as well as their symptom reduction, thereby potentially minimizing or exaggerating group differences.

The results of the present study suggest that a single measurement of the therapeutic alliance in an early phase of therapy is a good indicator of therapy outcome in patients with OCPD. In patients with BPD, one-time measurements of the therapeutic alliance appear to have limited predictive validity regarding therapy outcome. However, our results should by no means be understood as suggesting that the therapeutic alliance is of little importance for the outcome of therapy amongst BPD patients. In fact, the opposite might be true, especially when considering the high drop-out rate in BPD patients.

Based on our findings and seeing that a high rate of therapy discontinuation amongst BPD patients has frequently been observed in prior research and that a link between therapy discontinuation and helping alliance has been suggested (44, 45), we recommend more continuous measurements of alliance for BPD patients. In patients with OCPD, on the other hand, a one-time early measurement of the therapeutic alliance can provide valuable information about possible conflicts. These conflicts are often concealed or carried out passively in patients with OCPD, thereby making it more difficult for the therapist to detect them. Unspoken latent conflicts or dissatisfaction in the therapeutic alliance could thus be identified, addressed and resolved at an early stage. Furthermore, the fact that we found an exceptionally strong alliance-outcome relationship in OCPD patients compared to studies with a broader patient population suggests that a focus on the therapeutic alliance in therapy planning or intervention selection might be particularly beneficial in this patient group.

Taking account of the nature of interactional problems, it appears that those of BPD patients automatically push themselves into the foreground of the therapeutic situation, whereas the interactional difficulties of OCPD patients are more quiet in nature and often hidden under a coat of outward conformity and sense of duty. Our study points to the importance of not overlooking these interactional problems, in particular with regard to how they may impact key impact factors of therapy outcome.

In light of the above-mentioned limitations and the overall exploratory nature of the study, interpretations and implications should be considered tentatively. Further studies investigating the alliance-outcome association in different patient groups and settings are needed to obtain a more differentiated picture of the mechanisms of change in psychotherapy.

## Data availability statement

The raw data supporting the conclusions of this article will be made available by the authors, without undue reservation.

## Ethics statement

The studies involving human participants were reviewed and approved by the Ethics Committee of the Technische Universität Dresden (EK494122016). The patients/participants provided their written informed consent to participate in this study.

## Author contributions

PB and RN made substantial contributions to the conception and design of the present study. AK and RN were responsible for the acquisition of data, whereas PB and SS contributed substantially to the analysis and interpretation of data. PB took the lead in writing the manuscript, while RN was supervising the project. SS, RN, and KW revised the manuscript critically for important intellectual content. All authors gave final approval of the version to be submitted and any revised version.

## References

[1] Bordin ES. The generalizability of the psychoanalytic concept of the working alliance. Psychother Theor. Res. Practice. (1979) 16:252–60. 10.1037/h0085885

[2] Flückiger C Del Re AC Wampold BE Horvath AO. The alliance in adult psychotherapy: a meta-analytic synthesis. Psychotherapy. (2018) 55:316–40. 10.1037/pst0000172 29792475

[3] Horvath AO Del Re AC Flückiger C Symonds D. Alliance in individual psychotherapy. Psychotherapy. (2011) 48:9–16. 10.1037/a0022186 21401269

[4] Martin DJ Garske JP Davis MK. Relation of the therapeutic alliance with outcome and other variables: a meta-analytic review. J Consult Clin Psychol. (2000) 68:438–50. 10.1037/0022-006X.68.3.438 10883561

[5] Barber JP. Toward a working through of some core conflicts in psychotherapy research. Psychother Res. (2009) 19:1–12. 10.1080/10503300802609680 19206018

[6] DeRubeis RJ Brotman MA Gibbons CJ. A conceptual and methodological analysis of the nonspecifics argument. Clin Psychol Sci Prac. (2005) 12:174–83. 10.1093/clipsy.bpi022

[7] Kazdin AE. Treatment outcomes, common factors, and continued neglect of mechanisms of change. Clin Psychol Sci Prac. (2005) 12:184–8. 10.1093/clipsy.bpi023

[8] Strunk DR Cooper AA Ryan ET DeRubeis RJ Hollon SD. The process of change in cognitive therapy for depression when combined with antidepressant medication: predictors of early intersession symptom gains. J Consult Clin Psychol. (2012) 80:730–8. 10.1037/a0029281 22774791 PMC3458155

[9] Falkenström F Granstrom F Holmqvist R. Therapeutic alliance predicts symptomatic improvement session by session. J Couns Psychol. (2013) 60:317–28. 10.1037/a0032258 23506511

[10] Zilcha-Mano S Dinger U McCarthy KS Barber JP. Does alliance predict symptoms throughout treatment, or is it the other way around? J Consult Clin Psychol. (2014) 82:931–5. 10.1037/a0035141 24274627 PMC4032804

[11] Xu H Tracey TJG. Reciprocal influence model of working alliance and therapeutic outcome over individual therapy course. J Couns Psychol. (2015) 62:351–9. 10.1037/cou0000089 25985186

[12] Flückiger C Rubel J Del Re AC Horvath AO Wampold BE Crits-Christoph P. The reciprocal relationship between alliance and early treatment symptoms: a two-stage individual participant data meta-analysis. J Consult Clin Psychol. (2020) 88:829–43. 10.1037/ccp0000594 32757587

[13] Marker CD Comer JS Abramova V Kendall PC. The reciprocal relationship between alliance and symptom improvement across the treatment of childhood anxiety. J. Clin. Child Adol. Psychol. (2013) 42:22–33. 10.1080/15374416.2012.723261 23009693 PMC4224949

[14] Zilcha-Mano S Errazuriz P. One size does not fit all: examining heterogeneity and identifying moderators of the alliance-outcome association. J Couns Psychol. (2015) 62:579–91. 10.1037/cou0000103 26376176

[15] Lorenzo-Luaces L DeRubeis RJ Webb CA. Client characteristics as moderators of the relation between the therapeutic alliance and outcome in cognitive therapy for depression. J Consult Clin Psychol. (2014) 82:368–73. 10.1037/a0035994 24547921 PMC4063527

[16] Lorenzo-Luaces L Driessen E DeRubeis RJ Van HL Keefe JR Hendriksen M. Moderation of the alliance-outcome association by prior depressive episodes: differential effects in cognitive-behavioral therapy and short-term psychodynamic supportive psychotherapy. Behav Ther. (2017) 48:581–95. 10.1016/j.beth.2016.11.011 28711109

[17] Nevid JS Ghannadpour J Haggerty G. The role of gender as a moderator of the alliance-outcome link in acute inpatient treatment of severely disturbed youth. Clin Psychol Psychother. (2017) 24:528–33. 10.1002/cpp.2025 27174625

[18] Flückiger C Del Re AC Horvath AO Symonds D Ackert M Wampold BE. Substance use disorders and racial/ethnic minorities matter: a meta-analytic examination of the relation between alliance and outcome. J Couns Psychol. (2013) 60:610–6. 10.1037/a0033161 23815630

[19] Zack SE Castonguay LG Boswell JF McAleavey AA Adelman R Kraus DR. Attachment history as a moderator of the alliance outcome relationship in adolescents. Psychotherapy. (2015) 52:258–67. 10.1037/a0037727 25822108

[20] Piper WE Ogrodniczuk JS Joyce AS. Quality of object relations as a moderator of the relationship between pattern of alliance and outcome in short-term individual psychotherapy. J Pers Assess. (2004) 83:345–56. 10.1207/s15327752jpa8303_15 15548470

[21] Zilcha-Mano S Lipsitz I Errazuriz P. When is it effective to focus on the alliance? Analysis of a within-client moderator. Cognit Ther Res. (2018) 42:159–71. 10.1007/s10608-017-9867-4

[22] Del Re AC Flückiger C Horvath AO Symonds D Wampold BE. Therapist effects in the therapeutic alliance–outcome relationship: a restricted-maximum likelihood meta-analysis. Clin Psychol Rev. (2012) 32:642–9. 10.1016/j.cpr.2012.07.002 22922705

[23] De Bolle M Johnson JG De Fruyt F. Patient and clinician perceptions of therapeutic alliance as predictors of improvement in depression. Psychother Psychosom. (2010) 79:378–85. 10.1159/000320895 20829649

[24] American Psychiatric Association. Diagnostic and statistical manual of mental disorders. 5th Edn. Washington, DC: American Psychiatric (2013).

[25] Lives WJ. An empirically-based classification of personality disorder. J Pers Disord. (2011) 25:397–420. 10.1521/pedi.2011.25.3.397 21699399

[26] Lingiardi V Filippucci L Baiocco R. Therapeutic alliance evaluation in personality disorders psychotherapy. Psychother Res. (2005) 15:45–53. 10.1080/10503300512331327047

[27] Zimmerman M Rothschild L Chelminski I. The prevalence of DSM-IV personality disorders in psychiatric outpatients. Am J Psychiatry. (2005) 162:1911–8. 10.1176/appi.ajp.162.10.1911 16199838

[28] Russell JJ Moskowitz DS Zuroff DC Sookman D Paris J. Stability and variability of affective experience and interpersonal behavior in borderline personality disorder. J Abnorm Psychol. (2007) 116:578–88. 10.1037/0021-843X.1 16.3.578 17696713

[29] Aaronson CJ Bender DS Skodol AE Gunderson JG. Comparison of attachment styles in borderline personality disorder and obsessive-compulsive personality disorder. Psychiatric Quarterly. (2006) 77:69–80. 10.1007/s11126-006-7962-x 16397756

[30] Skodol AE Gunderson JG McGlashan TH Dyck IR Stout RL Bender DS. Functional impairment in patients with schizotypal, borderline, avoidant, or obsessive-compulsive personality disorder. Am J Psychiatry. (2002) 159:276–83. 10.1176/appi.ajp.159.2.276 11823271

[31] Soeteman DI Verheul R Busschbach JJ. The burden of disease in personality disorders: diagnosis-specific quality of life. J Pers Disord. (2008) 22:259–68. 10.1521/pedi.2008.22.3.259 18540798

[32] Cain NM Ansell EB Simpson HB Pinto A. Interpersonal functioning in obsessive–compulsive personality disorder. J Pers Assess. (2015) 97:90–9. 10.1080/00223891.2014.934376 25046040 PMC4281499

[33] Melges FT Swartz MS. Oscillations of Attachment in Borderline Personality Disorder. Washington, DC: American Psychiatric. (1989). 10.1176/ajp.146.9.1115 2764169

[34] Sable P. Attachment, detachment and borderline personality disorder. Psychother Theor Res Prac Training. (1997) 34:171. 10.1037/h0087674

[35] Loranger AW. International personality disorder examination (IPDE). Assessment and diagnosis of personality disorders The ICD-10 international personality disorder examination. IPDE. (1997) 17:43–51. 10.1017/CBO9780511663215.005

[36] First MB Gibbon M Spitzer RL Benjamin LS Williams JB. Structured Clinical Interview for DSM-IV^®^ Axis II Personality Disorders SCID-II. Washington, DC: American Psychiatric (1997).

[37] Derogatis L. BSI. 18, Brief Symptom Inventory 18: Administration, Scoring, and Procedures Manual. Minneapolis, MN: NCS Pearson, Inc. (2000).

[38] Luborsky L. Principles of Psychoanalytic Psychotherapy: A Manual for Supportive-Expressive Treatment. New York, NY: Basic Books, Inc (1984).

[39] Nübling R Kraft M Henn J Kriz D Lutz W Schmidt J. Testing the psychometric properties of the helping alliance questionnaire (HAQ) in different health care settings. Psychother Psychosom Med Psychol. (2017) 67:465–76. 10.1055/s-0043-111083 28854445

[40] Cohen J. Statistical Power Analysis for the Behavioral Sciences. Illsdale, NJ: Academic Press. (1988).

[41] Sharf J Primavera LH Diener MJ. Dropout and therapeutic alliance: a meta-analysis of adult individual psychotherapy. Psychother Theor Res Prac Train. (2010) 47:637. 10.1037/a0021175 21198249

[42] Gersh E Hulbert CA McKechnie B Ramadan R Worotniuk T Chanen AM. Alliance rupture and repair processes and therapeutic change in youth with borderline personality disorder. Psychol Psychother Theor Res Prac. (2017) 90:84–104. 10.1111/papt.12097 27240265

[43] Zilcha-Mano S. Is the alliance really therapeutic? revisiting this question in light of recent methodological. Adv Am Psychol. (2017) 72:311–25. 10.1037/a0040435 28481579

[44] Johansson H Eklund M. Helping alliance and early dropout from psychiatric out-patient care. Soc Psychiatry Psychiatr Epidemiol. (2006) 41:140–7. 10.1007/s00127-005-0009-z 16372143

[45] Roos J Werbart A. Therapist and relationship factors influencing dropout from individual psychotherapy: a literature review. Psychother Res. (2013) 23:394–418. 10.1080/10503307.2013.775528 23461273

